# The Impact of Opioid Prescribing Limits on Drug Usage in South Carolina: A Novel Geospatial and Time Series Data Analysis

**DOI:** 10.3390/healthcare11081132

**Published:** 2023-04-14

**Authors:** Amirreza Sahebi-Fakhrabad, Amir Hossein Sadeghi, Eda Kemahlioglu-Ziya, Robert Handfield, Hossein Tohidi, Iman Vasheghani-Farahani

**Affiliations:** 1 Department of Industrial and Systems Engineering, North Carolina State University, Raleigh, NC 27606, USA; asahebi@ncsu.edu (A.S.-F.); asadegh3@ncsu.edu (A.H.S.); 2 Department of Business Management, Poole College of Management, North Carolina State University, Raleigh, NC 27695, USA; ekemahl@ncsu.edu; 3 SAS Institute Inc., Cary, NC 27513, USA; hossein.tohidi@sas.com (H.T.); iman.vasheghanifarahani@sas.com (I.V.-F.)

**Keywords:** prescription drug abuse, policy analysis, interrupted time series, spatiotemporal classification, ARIMAX, pharmaceutical supply chain

## Abstract

The opioid crisis in the United States has had devastating effects on communities across the country, leading many states to pass legislation that limits the prescription of opioid medications in an effort to reduce the number of overdose deaths. This study investigates the impact of South Carolina’s prescription limit law (S.C. Code Ann. 44-53-360), which aims to reduce opioid overdose deaths, on opioid prescription rates. The study utilizes South Carolina Reporting and Identification Prescription Tracking System (SCRIPTS) data and proposes a distance classification system to group records based on proximity and evaluates prescription volumes in each distance class. Prescription volumes were found to be highest in classes with pharmacies located further away from the patient. An Interrupted Time Series (ITS) model is utilized to assess the policy impact, with benzodiazepine prescriptions as a control group. The ITS models indicate an overall decrease in prescription volume, but with varying impacts across the different distance classes. While the policy effectively reduced opioid prescription volumes overall, an unintended consequence was observed as prescription volume increased in areas where prescribers were located at far distances from patients, highlighting the limitations of state-level policies on doctors. These findings contribute to the understanding of the effects of prescription limit laws on opioid prescription rates and the importance of considering location and distance in policy design and implementation.

## 1. Introduction

Each year, 11.8 million deaths are caused by illicit drug use, smoking, and alcohol consumption, which surpasses the total number of deaths from all forms of cancer and car accidents in the United States [1,2]. Illicit drugs, such as opioids, are banned under international drug control agreements [3]. In 2020, 91,799 drug overdose deaths occurred in the United States and the age-adjusted rate of overdose deaths increased by 31% from 2019 (21.6 per 100,000) to 2020 (28.3 per 100,000). This happened while the Centers for Disease Control and Prevention (CDC) had declared that prescription drug abuse, including misuse of opioid medications, is at epidemic levels [4]. The number of drug overdose deaths in South Carolina increased by 53% from December 2019 (1131 cases) to December 2020 (1734 cases), making it one of the states with the highest drug overdose death rates in the U.S. [5]. Drug overdose deaths in the United States are primarily caused by synthetic opioids, with the exception of methadone. In 2020, opioids were a factor in 68,630 overdose deaths, accounting for 75% of all drug overdose deaths [6].

Given the growing awareness of potential negative consequences associated with opioid analgesics (OAs), various measures have been taken by states to curb the inappropriate use of these medications [7]. These include requiring healthcare providers to consult state Prescription Drug Monitoring Programs (PDMP) or Risk Evaluation and Mitigation Strategies (REMS) [8], as well as the need for continuing medical education (CME) which is mandated by some states [9]. In more recent times, many states have implemented prescription limits on certain scheduled drugs, such as opioids, in an effort to reduce the quantity of these medications that are available and to address the issue of opioid abuse and overdose [10]. These restrictions, many of which were passed since January 2017, vary widely among states, so the specific details of the prescription limits and which drugs are subject to the limits can differ depending on the location [11]. In May 2018, South Carolina implemented limits on the quantity of opioid prescriptions that can be issued by healthcare providers. Under this law, the maximum supply of opioids that can be prescribed is generally limited to a five-day supply or a daily maximum of 90 morphine milligram equivalents (MMEs), unless the patient is receiving treatment for chronic pain, cancer pain, pain related to sickle cell disease, hospice care, palliative care, or medication-assisted treatment for substance use disorder (S.C. Code 44-53-360 section j) [12]. This law is solely designed for opioid drugs and does not include other controlled substances like benzodiazepine.

Measuring the effects of these policies in healthcare systems, enhancing system performance, and improving the quality of evaluators and their tasks are critical components of the evaluation [13]. Studies on state-implemented policies to combat opioid misuse and overdose have produced inconsistent results [14]. While some studies suggest that policies such as dosage limits have reduced overdose deaths [15], others indicate that prescription drug monitoring programs have only had a small effect on opioid shipments [16] and that these laws are not very effective [17]. Research on the effectiveness of opioid limit laws enacted mostly after 2017 is also limited, and analysis of their impact on the entire opioid supply chain, including prescribers and dispensers, is lacking in the literature [10]. Furthermore, no studies have evaluated the unintended impacts of these policies on suspicious behaviors, such as traveling long distances to obtain opioids.

To address this gap, this paper presents a comprehensive analysis of opioid policies and stakeholders in South Carolina. A spatiotemporal classification system is proposed that considered the locations of and distances between prescribers, dispensers, and patients, as well as the total distance traveled by patients. The system is used to test two hypotheses: The system is used to test two hypotheses: (1) the total distance traveled by the patient affects the prescription volume, and individuals who travel longer distances to obtain opioid prescriptions may have a higher chance of misusing or abusing these drugs, leading to higher dosage (H1); (2) the relative location of doctors and pharmacies plays a crucial role in policy effectiveness and prescription volume, particularly among patients who travel longer distances (H2).

To assess the effectiveness of the opioid prescription limit law implemented in South Carolina in May 2018, we use interrupted time series models to compare the pre-policy and post-policy data while considering trends and correlations. To evaluate the potential drawbacks of the policy on different groups, we implement the time series models at the level of each group in the classification system. We also use benzodiazepine prescriptions as a control group to validate our results.To the best of our knowledge, no other research has used clustering methods based on distance to analyze the impact of opioid policies.

The subsequent sections of this paper will outline the research design, data, and statistical methods employed. In Section 2, we will provide a comprehensive overview of the research design, including the data and statistical techniques utilized. Section 3 will exhibit the descriptive statistics of the classes in the proposed classification system, as well as examine the effect of the policy using ARIMAX models. Finally, the results will be summarized and potential avenues for future research will be recommended in Section 4 and Section 5.

## 2. Methodology

### 2.1. Research Design

We first develop a classification system based on the location of and the distance between stakeholders in prescription records to identify patterns, trends, and potentially suspicious activity in the opioid supply chain. Specifically, we test if there are statistically significant differences between the the average MMEs across the different groups in the classification. We then use an interrupted time series quasi-experimental design to evaluate the impact of a prescription limit policy on opioid prescribing practices in South Carolina. This approach allows us to examine the policy’s effects on different subgroups within the population, such as the location of each player (patient, prescriber, and dispenser), and understand the overall impact on opioid prescribing practices in the state. This approach is used in prior research such as a study that evaluates the impact of an opioid reduction act enacted in West Virginia in June 2018 [18], another study that uses ARIMA models to analyze the effects of a North Carolina medical board initiative to reduce high-dose and high-volume opioid prescribing as well as legislation to limit initial opioid prescriptions for acute and post-surgical pain [19]. The impact of Florida PDMP implementation on oxycodone-caused deaths was evaluated using the same approach as in [20]. To control for temporal trends in prescribing of similar medications, we also examine the volume of incident benzodiazepine prescriptions as a controlled substance that is not expected to be affected by the opioid policies as stated in other researches.

### 2.2. Data Description

**Figure 1:**
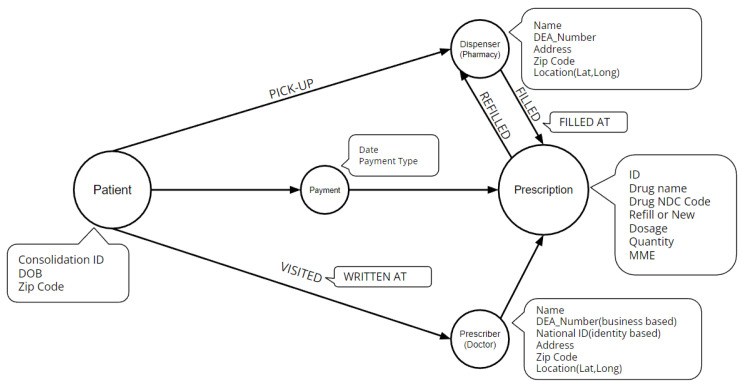
Metagraph of the SCRIPTS dataset.

We use data obtained from South Carolina Reporting and Identification Prescription Tracking System (SCRIPTS). The database contains 43 million prescription records from 2014 to 2022, including information about patients, prescribers, and dispensers for each particular opioid prescription. The interactions between them are analyzed using a network representation in Figure 1.

The data has the following information:

- ***Drug Information***
  – *National Drug Code (NDC)*: which is a unique identifier given by the FDA to each medication sold in the United States. It is used to identify and track drugs through the supply chain from manufacturer to pharmacy. The NDC is a 11-digit number composed of three segments, which include the product code, package code, and labeler code. These segments respectively identify the active ingredients and strength of the medication, the package size and type, and the manufacturer or distributor of the medication.
  – *Drug Name*: which is used to classify each drug further. For the purpose of our study, we group drugs into benzodiazepine (16% share) and opioids (84% share). We also classify opioid drugs further into fentanyl (2% share), hydrocodone (50% share), hydromorphone (1% share), methadone (1% share), morphine (4% share), oxycodone (25% share), oxymorphone (less than 1% share), and tapentadol (1% share).
  – *Drug Volume*: This is quantified by the quantity of drugs and by using the Morphine Milligram Equivalent (MME) to standardize the measurement of opioid dosage across different medications. MME is a unit of measurement that expresses the total daily dose of opioids in terms of an equivalent dose of morphine. It is used to assess the risk of overdose and other adverse events, to monitor opioid prescribing practices, and to track trends in opioid use over time. The MME is calculated by converting the total daily dosage of each opioid to an equivalent dose of morphine using conversion factors determined by the FDA based on the relative potencies of the different opioids.
- ***Prescription Information***
  – *Days Supply*: which refers to the number of days that a prescription for a specific medication will last, assuming average usage, which is calculated by dividing the total quantity of the medication prescribed by the average daily dosage. This measure is important in medication management, ensuring patients have enough medication and preventing overuse or misuse.
  – *Refill Status*: which refers to whether a prescription is for a new medication or a refill of a previously prescribed medication. A new medication prescription is written when a patient starts a new medication for the first time, while a refill prescription is a request to renew a previously filled prescription. It’s important to know the new or refill status because it can affect the dispensing process, patient’s insurance coverage and prescription regulations. Among all records 87% of them are new and only 13% are refilled.
  – *Payment Type*: which refers to the source of funds used to pay for the medication, the most common types being private insurance (36% share), Medicare (16% share), Medicaid (4% share) and other types with less than 2% share. It’s noteworthy to mention that 34% of the records have no available payment method.
  – *Date*: Each prescription has two important dates: the date the prescription was written by the prescriber, and the date the prescription was filled at the pharmacy.
  – *Authorized Refill Count*: which refers to the number of times a prescription can be refilled as authorized by the prescribing healthcare provider. It is specified on the prescription and can be used by pharmacies to track remaining refills.
- ***Prescriber Information***
  – *Location*: which includes full address of the prescriber.
  – *ID*: For healthcare providers, there are two types of identification numbers: National Provider Identifier (NPI) and Drug Enforcement Administration (DEA) number. NPI is assigned by the Centers for Medicare and Medicaid Services (CMS) to identify healthcare providers in transactions such as submitting claims, ordering or prescribing medication, or referring patients to other providers. DEA number is assigned by the DEA to track and regulate the prescribing and dispensing of controlled substances, it is a requirement for healthcare providers to have a DEA number in order to prescribe controlled substances. In summary, NPI is used for administrative purposes and DEA number is used for regulatory purposes.
- ***Dispenser Information***
  – *Location*: which includes full address of the dispenser.
  – *ID*: Dispensers have only one ID which is their DEA number.
- ***Patient Information***
  – *Consolidation ID*: Pre-assigned id in order to identify each patient.
  – *Age*.
  – *Location*: Which includes patient’s zip code.

### 2.3. Classification System

#### 2.3.1. Description

Prior research has established a clear link between traveling long distances and illicit behavior, such as doctor shopping, in obtaining opioid drugs. For instance, a study conducted on individuals who sought ADHD medication revealed that those who engaged in doctor shopping tended to travel longer distances, visit more states, and use cash payments more frequently compared to non-shoppers [21]. Similarly, another study on opioid prescription filling found that shoppers traveled longer distances, frequently crossed state borders, and were responsible for a significant portion of opioid dispensing [22]. These results highlight the significance of distance as a factor in identifying potential opioid-related misconduct. However, there is currently a lack of research on how distance impacts the entire distribution process of opioids, and how opioid policies can help address the challenges associated with long-distance travel for obtaining opioids.

In light of these research findings, we introduce a classification system that grouped prescription records based on the location of stakeholders (patients, prescribers, and dispensers) and the total distance traveled (indicated as π) to fill the prescription in the state of South Carolina. The system was divided into four distance groups: less than 250 miles, between 250 and 500 miles, between 500 and 1000 miles, and over 1000 miles, and three disparity groups: patient isolated, prescriber isolated, dispenser isolated.

The distance intervals are chosen based on two key observations, with the second being particularly significant. The first observation pertains to the average distance from the center of South Carolina to its border, which is approximately 250 miles. Transactions with a total distance of fewer than 500 miles (round-trip) fall within the state’s border, while distances between 500 and 1000 miles fall within the borders of neighboring states. However, the other observation is more critical: the average distance patients travel is 101.73, with a standard deviation of 143.64. To detect abnormalities in the data, intervals of 250, 500, and 1000 are used, representing one standard deviation, three standard deviations, and almost six standard deviations from the mean, respectively. These intervals provide an effective framework for identifying outliers and anomalies in the data, which is essential in ensuring accurate and reliable results. While more complex classifications with smaller intervals based on distance are possible, they may not be practical in terms of the model’s interoperability. Therefore, the current classification system strikes a balance between the need for granularity and the practicality of implementing a user-friendly model.

The disparity groups are underpinned by a second logic, which highlights the typical proximity of two or three out of the three stakeholders. For instance, it is common to observe a scenario where a patient visits a doctor, receives a prescription, and fills it in a nearby pharmacy on the way home. In contrast, it is uncommon to witness a pattern where a patient visits doctors or pharmacies located far away from each other. This classification system is particularly useful for detecting patterns and trends in the opioid prescription supply chain.

We hypothesize that this system can help identify patterns and trends in the opioid prescription supply chain. Specifically, we propose two hypotheses for further analysis using the proposed classification system.

Our first hypothesis, $H_{1}$, is that the total distance traveled by the patient affects the prescription volume, and individuals who travel longer distances to obtain opioid prescriptions may have a higher chance of misusing or abusing these drugs, leading to higher dosage. By analyzing the correlation between distance and prescription volume, we can better understand how distance affects opioid abuse and identify potential solutions to address the problem.

Our second hypothesis, $H_{2}$, is that the relative location of doctors and pharmacies plays a crucial role in policy effectiveness and prescription volume, particularly among patients who travel longer distances. This hypothesis is based on previous research findings that suggest unintended consequences of policies on out-of-state doctors and pharmacies [23].

Table 1 displays the classification system utilized in our study, while the result Section 3 presents the outcomes of our hypothesis testing. In the subsequent sections, we will elaborate on the methodology employed in applying this classification system to our dataset.

#### 2.3.2. Graph Model Definition

The graph, represented as G=(V,E), is made up of three sets of nodes: patient ($V_{1}$), prescribers ($V_{2}$), and, dispensers ($V_{3}$). The total number of nodes in the graph is *n* which is the sum of the number of patients, prescribers, and dispensers. The edge set, *E*, is a subset of V×V and represents connections between nodes. The focus of this research is on undirected attributed networks. The attribute matrix, *X*, is a matrix with *n* rows and *D* columns, where each row, $x_{v}$, represents the D-dimensional attribute vector for node (*v*).

***Neighbors.*** A vertex *u* is considered to be a neighbor of another vertex *v* in the graph *G* if there is a transaction recorded between them in the dataset. This is also referred to as *u* being adjacent to *v*.

**Figure 2:**
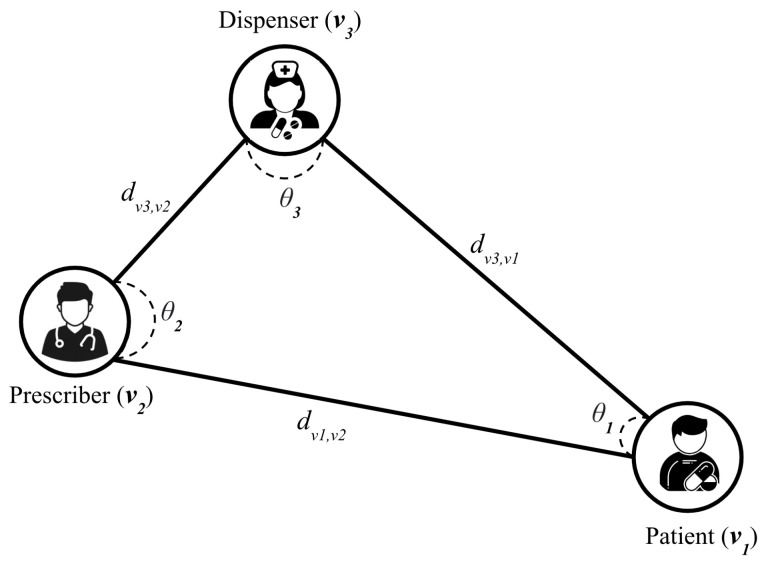
The location-based classification system of three stakeholders in the opioid supply chain.

***Transaction Cycle.*** In the graph, a transaction cycle is defined as a path that starts and ends at the same vertex, does not have any repeated vertices other than the starting and ending vertex, and has a length of three that includes at least one vertex from each of the three sets ($v_{1}$, $v_{2}$, $v_{3}$). T is a set of such transaction cycles and $\Omega_{v}$ is an attribute matrix with |T| rows and *D* columns, where each row $\omega_{v}$ represents the D-dimensional attribute vector for a specific transaction cycle (ω). In other words, each transaction cycle in the dataset is represented as a triangle (Figure 2).

This study focuses on two key attributes of each transaction cycle: distance-level and disparity-level representations, which are learned using a new degree-specific graph network model. Additionally, the paper examines the proposed model from various perspectives, including the drug usage volume measured by MME before and after policy implementation.

Formally, the distance-level and disparity-level representation learning problems can be defined as follows:

***Definition: (Distance-Level Representation)***

**Input**: Zip-code, represented as a node attribute *X* of graph *G*, where each $x_{v}$ has a latitude and longitude value in the matrix *X*.

**Output**: $\pi_{t}$; The perimeter of the triangle formed by the three nodes of a transaction cycle.

(1) $$d_{u,v}=2R.arcsin\left(\sqrt{sin^{2}\left(\frac{lat_{u}-lat_{v}}{2}\right)+cos\left(lat_{u}\right).cos\left(lat_{v}\right).sin^{2}\left(\frac{long_{u}-long_{v}}{2}\right)}\right)$$

(2) $$\pi_{t}=d_{v_{1},v_{2}}+d_{v_{1},v_{3}}+d_{v_{2},v_{3}}\;\forall t\in T$$

Formula (1) calculates the geodesic distance between any two nodes in the transaction cycle. We then sum them up to obtain the perimeter of the triangle representing the transaction cycle using formula 2. Where $v_{1}$,$v_{2}$,$v_{3}$ are the nodes of the transaction cycle, and the attributes (lat, long) represent the corresponding latitude and longitude of the nodes.

***Definition. (Disparity-Level Representation)***

**Input**: Zip-code, represented as a node attribute *X* of graph *G*, where each $x_{v}$ has a latitude and longitude value in the matrix *X*.

**Output**: $\theta_{i}$; interior angles of node $v_{i}$ in the triangle

To compute the interior angles of a triangle representing the transaction cycle, the paper first use the geodesic distance between the nodes in the transaction cycle ($d_{v_{i},v_{j}}$) as computed in the distance-level representation learning problem, then calculate the angles by using the dot product of the normalized distances between the node and two other nodes in the transaction cycle.

(3) $$\theta_{i}=arccos\left(\frac{d_{v_{i},v_{j}}}{|d_{v_{i},v_{j}}|}.\frac{d_{v_{i},v_{k}}}{|d_{v_{i},v_{k}}|}\right)\;\forall i\in V$$

#### 2.3.3. Classification Algorithm

The algorithm (Algorithm 1) presented in the study, based on the previously defined network, classifies each transaction t∈T into $\omega_{t}=\left(C\left[1\right],C\left[2\right]\right)$ code based on the disparity and distance of the stakeholders involved. The attributes of each transaction are split into three levels, and these levels are used to create a two-dimensional code, where C[1] represents the disparity level and C[2] represents the distance level.

### 2.4. Statistical Analysis

#### 2.4.1. Dependent Variable

In this study, the daily MME per prescription is the dependent variable used as a metric for drug volume. This measure was chosen over others for two reasons. Firstly, it aligns with the prescription limit law being studied, which sets limits on opioid prescriptions based on daily MME and days supply. Secondly, the CDC has classified the hazard of prescriptions based on the MME per day. The CDC’s classification includes four levels of MME/day dosage, with respective hazard ratios of 1, 1.44, 3.73, and 8.87, for levels 1, 2, 3, and 4, respectively [24,25]. According to CDC guidelines, prescriptions with more than 90 MME/day should be avoided, and those with more than 50 MME/day should be carefully assessed. The daily dosage is calculated by dividing the total MME by the number of days the prescription is intended to last, as indicated by the “days supply” field in the SCRIPTS data set. Prescriptions with missing days supply data were excluded from the analysis.
**Algorithm 1** Two-digit coding classifier  1:(Phase I: Classify based on distance level)  2:**Input:**  3:π← Perimeter of the triangle  4:$\Theta_{v}\leftarrow$ Angle of the node *v*  5:**Output: **ω  6:**if **π≤250** then**  7:   C[1]←0  8:**else if **π≤500** then**  9:   C[1]←110:**else if **π≤1000** then**11:   C[1]←212:**else**13:   C[1]←314:**end if**15:(Phase II: Classify based on distance-level)16:**for **(i,j)∈{(x,y)|x∈V,y∈V,x≠y}** do**17:   $r_{i,j}=\frac{\theta_{i}}{\theta_{j}}$18:**end for**19:**if **$r_{1,2}<0.5\wedge r_{1,3}<0.5\wedge \left(\left(r_{2,3}<1.25\wedge r_{2,3}>0.75\right)\vee \left(r_{3,2}<1.25\wedge r_{3,2}>0.75\right)\right)$** then**20:   C[1]←021:**else if **$r_{2,1}<0.5\wedge r_{2,3}<0.5\wedge \left(\left(r_{1,3}<1.25\wedge r_{1,3}>0.75\right)\vee \left(r_{3,1}<1.25\wedge r_{3,2}>0.75\right)\right)$** then**22:   C[1]←123:**else if **$r_{3,1}<0.5\wedge r_{3,3}<0.5\wedge \left(\left(r_{1,1}<1.25\wedge r_{1,3}>0.75\right)\vee \left(r_{2,1}<1.25\wedge r_{2,3}>0.75\right)\right)$** then**24:   C[1]←225:**else**26:   C[1]←327:**end if**28:**for **t∈T** do**29:   $\omega_{t}\leftarrow \left(C\left[1\right],C\left[2\right]\right)$30:**end for**31:**Return: **$\omega_{t}$

#### 2.4.2. Independent Variables

*Prescription Limit Law*: South Carolina has implemented policies to address opioid misuse and abuse. The state’s Code of Laws, Title 44, relates to health, and Chapter 53 specifically addresses controlled substances, including narcotics. Article 3 of the chapter pertains to narcotics and controlled substances. One specific policy, Subsection J of S.C. Code 44-53-360, limits initial opioid prescriptions for acute or postoperative pain management to a seven-day supply or 90 morphine milligram equivalents daily, except in certain circumstances. These include cancer pain, chronic pain, hospice care, palliative care, major trauma, major surgery, sickle cell disease treatment, neonatal abstinence syndrome treatment, or medication-assisted treatment for substance use disorder. Subsequent consultations allow for appropriate renewal, refill, or new opioid prescriptions. The law does not apply to prescriptions wholly administered in hospitals, nursing homes, hospice facilities, or residential care facilities. The focus of our study is on the impact of this policy, which is the most recent implemented and directly affects prescription dosage. We use a binary indicator variable (0 = pre-implementation, 1 = post-implementation) to model the policy’s impact.

*Internal Control Series*: To serve as a control group in the study, a dataset of benzodiazepine prescriptions was used because there is similar pressure to reduce benzodiazepine prescriptions, even though they were not specifically targeted in the policy. It is worth noting that the state of South Carolina has not yet imposed any restrictions on the volume of benzodiazepine prescriptions. Furthermore, although the CDC recommends avoiding the use of benzodiazepines with opioid medications, they were not explicitly mentioned in the policy. The dataset included all benzodiazepines dispensed by pharmacies in the state, such as alprazolam and chlordiazepoxide. In addition, because benzodiazepines have a lower potential for addiction, they were an appropriate option for comparison purposes and used in previous researches as well [18,19,26].

#### 2.4.3. One Way ANOVA

To evaluate the usefulness of the classification model and compare different classes, we will employ one-way ANOVA. This statistical test is utilized to measure the difference between groups by obtaining samples from each group, computing the within-sample, between-sample, and sample size, and aggregating all information into the F-value. The test’s effectiveness relies on certain assumptions, including group independence and data normality. ANOVA is considered a valid approach when dealing with large sample sizes, and deviations from normality are not a significant concern. Homogeneity of variances is also critical, especially in the case of an unbalanced design, which can be verified using Levene’s test. If significant differences are discovered through ANOVA, a post hoc test like Tukey can be utilized to determine which group means differ significantly [27].

#### 2.4.4. Interrupted Time Series Analysis

Causal inference is a set of widely recognized statistical techniques used to examine the effect of an intervention, such as the adoption of a policy. This field of study aims to comprehend the relationship between cause and effect by examining how alterations in one variable affect changes in another. This is achieved through two types of designs: experimental and quasi-experimental. In experimental designs, the intervention is randomly assigned to units in the population, allowing for a comparison of outcomes between affected and unaffected units [28]. However, there may be circumstances where randomized trials cannot be performed, such as when the intervention affects an entire country or has historical implications [29]. In such cases, quasi-experimental designs are used, where the treatment group is formed based on criteria such as time, location, or existing characteristics, while the control group is usually formed in a similar manner but does not receive the intervention [28].

Interrupted Time Series (ITS) is one of the most well-known quasi-experimental methods for intervention analysis. ITS is a more elaborate version of the segmented regression that divides the longitudinal data of the study into pre-policy and post-policy segments, and performs regression analysis on these sections rather than the entire time period. As previously mentioned in the literature, pre-policy trends have the potential to affect the results in a biased manner. Therefore, it is essential to take into account the trends and seasonal patterns in the pre-policy data. ITS is a robust design that controls for these pre-policy trends by tracking the outcome over time both before and after the intervention. It is considered one of the best designs for establishing causality and is often as reliable as randomized controlled trials (RCTs) when used with a control series [30]. Modeling data from the pre-policy period provides insight into the underlying trend, which, when extrapolated into the post-policy period, generates an estimate of what would have occurred without the intervention. By comparing this estimate with the actual observations post-policy, one can determine the impact of the intervention, taking into account the underlying trend. This method enables the calculation of both immediate and long-term effects [31].

The ARIMA method, which was first introduced in 1976, is a popular technique for analyzing ITS models. It merges elements from both autoregressive (AR) and moving average (MA) models to predict stationary and non-stationary data. In an AR model, the predicted variable is based on its own prior values and an error term, while in an MA model, the predicted variable relies on current and previous values of random shock terms. The ARIMA model combines these two components and uses differencing to make the data stationary. It has the following form:

(4) $$y_{t}^{\prime }=c+\left(\phi_{1}y_{t-1}^{\prime }+\ldots +\phi_{p}y_{t-p}^{\prime }\right)+\left(\theta_{1}\epsilon_{t-1}+\ldots +\theta_{q}\epsilon_{t-q}\right)+\epsilon_{t}$$

where *c* is a constant, $Y_{i}$ is time series value at time *i*, $\phi_{1}$, $\phi_{2}$, …, $\phi_{p}$ are parameters of the AR model with *p* lags, $\epsilon_{t}$ is normal random noise at time *t*, $\theta_{1}$, $\theta_{2}$, …, $\theta_{q}$ are coefficients of the MA model with *q* orders, and $y_{t}^{\prime }=\Delta^{d}y_{t}$ is the $d^{th}$ order difference of $y_{t}$ helps to produce a stationary process.

The following steps were taken to incorporate ARIMA in ITS:

1. An ARIMA model was fitted for each time series under study, specifically for the pre-intervention period. The pre-intervention period is considered the period before the policy enactment in May 2018.
2. An autoregressive integrated moving average with exogenous variables (ARIMAX) model was estimated for the entire time series by using the best ARIMA orders found in the previous step, adding regressor variables corresponding to the announcement and implementation of the legislation.
3. All previous steps were repeated for both subgroups proposed by the classification system and for the aggregated data without any subgroup.

This approach has been previously reported in the literature [18,19,20,32,33]. The Automatic Time Series Modeling (ATSM) package of SAS Visual Forecasting software (Viya 4—Cadence version 2022.12) was used to find the best ARIMA models for each series [34]. The software automatically detects the required differencing orders by running simple and seasonal augmented Dickey-Fuller tests. Then, it finds the tentative autoregressive moving average (ARMA) orders using the minimum information criterion. Next, the optimizer finds the best ARMA orders, bounded by their respective tentative values, considering the Bayesian information criterion (BIC). The seasonal ARMA orders are similarly detected while they are less than or equal to 2. The intervention effect on the response variable was analyzed by adding event inputs in shape of level-shift, ramp and inverse trend to the final ARIMA model for each series through the interactive modeling node of SAS Visual Forecasting [35].

## 3. Results

### 3.1. Opioid Usage in the Different Groups in the Spatial Classification

To verify the hypotheses presented in Section 2.3.1, we implemented the classification system on the SCRIPTS dataset. We excluded data before 2014 due to the high level of noise. Additionally, we removed prescriptions with an unusual MME value exceeding $10^{5}$ from the dataset. The data was then consolidated at the monthly level to allow for time series analysis in the following section. This decision was made because data consolidation at any other level would have resulted in intermittent and non-stationary data, which are not compatible with ARIMA models. To provide a summary of key statistics, we calculated the days of supply, MME, MME per day (determined by dividing MME by the number of days of supply), and class percentage (calculated by dividing the total number of records within a specific group by the total number of records in the dataset) for opioid drugs. These statistics are presented in Table 2.

According to the results, the majority of transactions (over 90%) have a traveled distance of less than 250 miles, which suggests that most transactions are not suspicious in terms of distance. The remaining transactions fall into the “1X”, “2X”, and “3X” distance categories, with each accounting for around 3% of the data. It is important to note that having only a few transactions in these categories is not a limitation of the classification system, but rather it may reveal unusual prescription patterns with low frequency. This information may not be detectable when aggregated with other groups and can provide valuable insights into the nature of the transactions.

In terms of dosage which is measured by daily MME, the average is 46.85 with a standard deviation of 9.29, indicating a 37% probability that it is above 50. Dosages above 50 MME should be closely evaluated, and some classes have a daily MME level exceeding 50 or even 90. Among the defined classes, five of them have an average MME/day of more than 50. Higher MMEs are mostly found in groups where patients travel more than 500 miles to obtain their drugs from distant pharmacies, increasing the likelihood of suspicious activities. These observations support the first two hypotheses.

**Figure 3:**
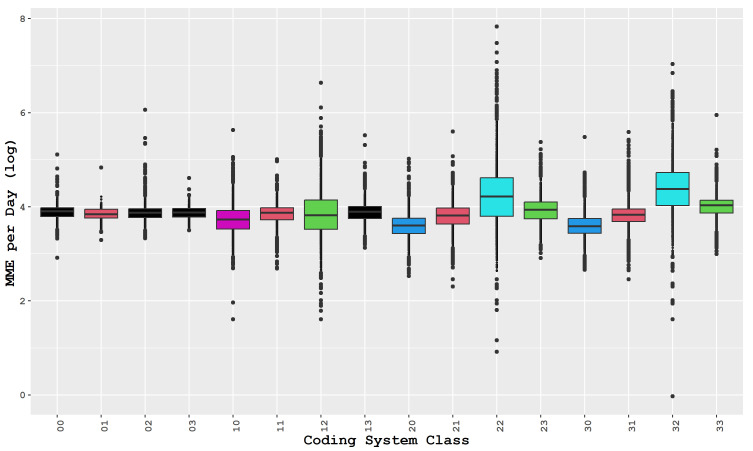
MME per day box-plot with Tukey’s results for opioid drugs. Classes with overlapping confidence intervals are plotted using the same color.

To test the first hypothesis, we assessed the effectiveness of the introduced classification system. If there were no differences between the classes, then the system would be meaningless, and we would not be able to relate distance to prescription volume and patterns. We employed one-way ANOVA to determine the difference between the groups and assumed independence and normality based on our data’s nature, and the sample sizes were sufficient to support this assumption. We used Levene’s test to test the final assumption of homogeneity of variances, which indicated that the assumption of variance equality was invalid (with a *p*-value of $2.26\times e^{-16}$). Therefore, we proceeded with Welch’s ANOVA, which also rejected the equality of coding classes mean (with a *p*-value of $2.26\times e^{-16}$), supporting our hypothesis that the proposed system can identify differences among groups and that distance is a crucial factor in determining prescription patterns. We performed a Tukey HSD post-hoc analysis to identify the groups that exhibit significant differences. Figure 3 displays the results, with paired classes sharing the same color. Based on the figure, it can be seen that groups 22 and 32 display significant differences from the other groups. In total, there are six groups of classes identified based on their respective colors.

The misuse and diversion of opioids continue to pose a complex problem, and its source remains inconclusive. However, studies on medically prescribed opioids suggest that diversion mainly happens during the prescription phase [36]. Thus, the second hypothesis of this research aims to pinpoint the possible sources of misuse. Our study’s findings reveal that classes with prescription levels of over 50 or 90 MME per day are the ones with the highest total distance traveled and isolated pharmacies. We conducted a one-sided *t*-test at a 95% confidence interval, with the alternative hypothesis stating MME per day above 50 or 90, and the null hypothesis stating MME per day less than 50 or 90. Test shows that groups 12, 22, 23, 32, and 33 have prescription volumes above 50 MME per day, while all other classes are below 50 MME per day. Additionally, classes 22 and 32 have MME per day exceeding 90, which is particularly alarming. These results validate our second hypothesis and underscore the role of pharmacies.

In order to delve into the issue concerning groups 22 and 32, which are linked to distant pharmacies, we analyzed the pharmacies most commonly used in each class. The dataset comprises various types of pharmacies, with the top three being: (1) Chain (2) Retail (3) Mail-order. To gain a clearer understanding of these pharmacy categories, a brief description of each one is provided below:

1. **Chain pharmacies** are part of a larger corporation that operates multiple pharmacy locations. They offer a wide range of prescription and over-the-counter medications, health and wellness products, and services such as immunizations and health screenings.
2. **Retail pharmacies** are typically located within supermarkets, department stores, or other retail establishments. They offer a selection of medications, health and wellness products, and over-the-counter medications.
3. **Mail-order pharmacies** deliver medications directly to patients’ homes via mail or courier services. They are a convenient option for patients with chronic conditions who require regular medication refills.

**Figure 4:**
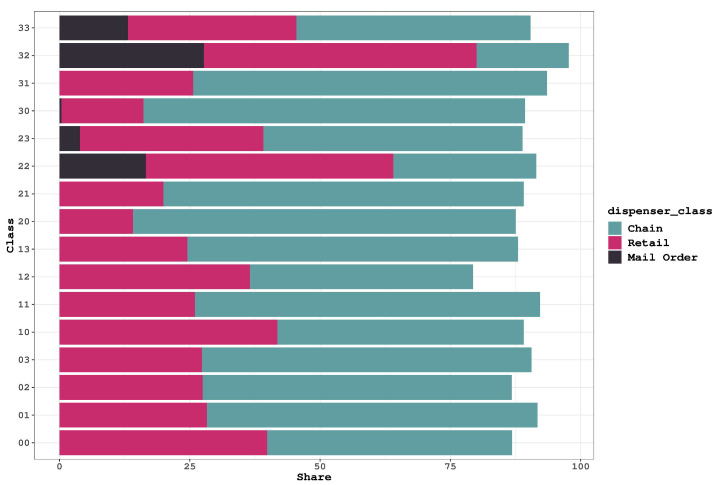
Pharmacy Distribution by Class: Chain and Retail Pharmacies Dominate, While Mail-Order Pharmacies Appear in Problematic Classes.

The distribution of pharmacies in each group is displayed in Figure 4. The data illustrates that chain pharmacies are the most common in the majority of classes, followed by retail pharmacies. However, in the four problematic classes (22, 23, 32 and 33), the dominant group consists of retail and mail-order pharmacies, with chains being less prevalent. The presence of mail-order pharmacies in the groups that attempt to obtain drugs from pharmacies located far away is not surprising. Nevertheless, the unusual prescriptions filled by these groups could indicate suspicious activities within them. Investigating this issue further is beyond the scope of this research and requires further attention. Thus far, substantial differences have been observed among the distant-based prescription classes. Therefore, we are interested in examining the effect of the prescription limit policy on these groups.

### 3.2. Policy Analysis Using Time Series Models

On 1 May 2018, the State of South Carolina put in place a prescription limit policy as a measure to combat the opioid epidemic. The policy aimed to restrict the number of opioid prescriptions that can be given to patients [37]. The policy’s impact was evaluated by analyzing data from SCRIPTS and comparing the volume of opioid prescriptions before and after the policy’s implementation. A simple pre-post policy comparison shows that on average, the MME per day was reduced by 2.59 (from 53.68 (95% CI [53.33, 54.02]) to 51.09 (95% CI [50.74, 51.44])) and the majority of groups in the proposed classification system experienced a significant reduction in the volume of prescribed opioids, except for groups 22 and 31 (as shown in Table 3). To study this observation rigorously, a combination of ARIMA and ARIMAX models, as a class of interrupted time series models, were used to further analyze the policy’s impact over time and how it has affected different groups.

**Figure 5:**
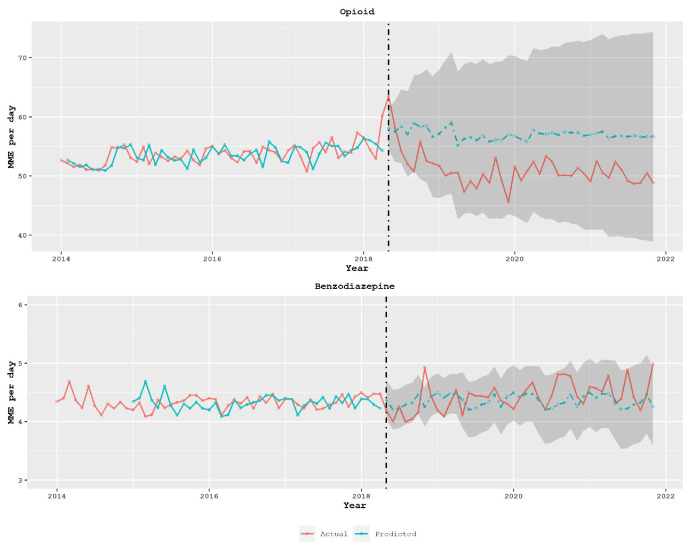
The top graph shows the average daily MME for opioid prescriptions in the state of South Carolina over a period of time (measured in months). The broken vertical line indicates the legislative intervention that was enacted in May 2018. The blue dotted line in the graph represents the fit of the mathematical model. The bottom graph demonstrates the same for benzodiazepine as the control group.

As a first step, an ARIMA model was applied to the pre-intervention data and used to predict the post-intervention prescription volume. The forecast results of the model, along with the 95% prediction confidence interval, are illustrated in Figure 5. The discrepancy between the predicted values and actual ones in the opioid data is an indication of the intervention’s impact. In the absence of any intervention effect, it would be expected that the pre-policy and post-policy data would follow the same pattern. Notably, the discrepancy is not present in the benzodiazepines, which serve as the control group.

**Figure 6:**
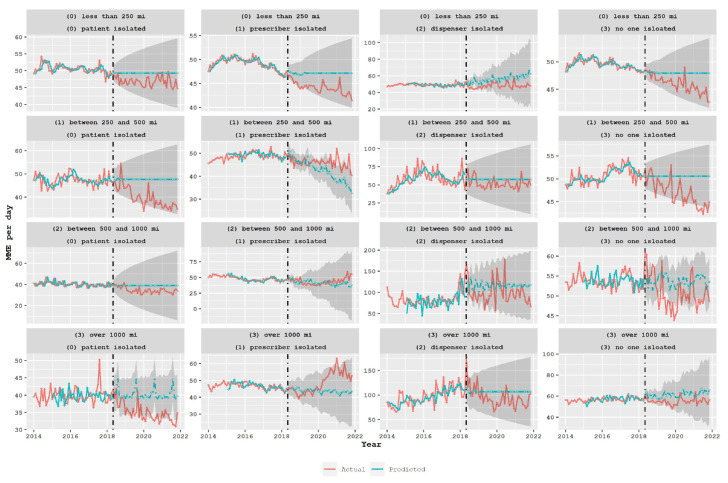
Average daily MME for opioid prescriptions in the state of South Carolina over a period of time (measured in months) for different proposed classes. The broken vertical line indicates the legislative intervention that was enacted in May 2018. The blue line represents the fit of the mathematical model.

**Figure 7:**
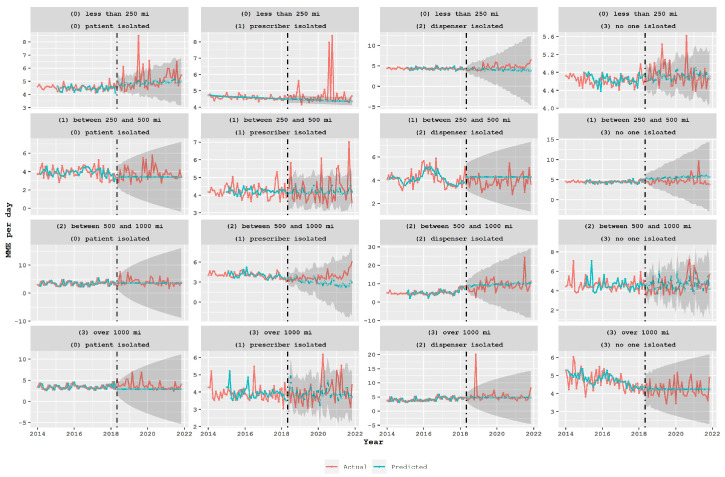
Average daily MME for benzodiazepine (control group) prescriptions in the state of South Carolina over a period of time (measured in months) for different proposed classes. The broken vertical line indicates the legislative intervention that was enacted in May 2018. The blue line represents the fit of the mathematical model.

The analysis was expanded to assess the impact of the policy on the various groups of the proposed classification system, as depicted in Figure 6. The desired mismatch, where actual values decrease while predicted values remain constant, was observed for groups with a total traveled distance of less than 250 miles (first row) and patient-isolated classes (first column). However, when examining the prescriber-isolated classes (second column), the mismatch was observed in the opposite direction, meaning that after the policy was adopted, predicted values were below the actual values, specifically in classes 11 and 31. This indicates that the policy had an impact, but in the opposite direction, encouraging patients to travel long distances to get drugs from distant doctors, which may lead to unintended consequences. In the dispenser-isolated classes (third column), there was a higher MME per day, but no reduction in post-policy predicted or actual values was observed, indicating no policy impact. In the last column, a reduction in actual values and mismatch was observed in groups 03 and 13. A similar analysis was performed for benzodiazepine prescriptions, and the results are presented in Figure 7. The policy did not appear to have a significant impact on the volume of benzodiazepine prescriptions, and the mismatch between predicted and actual values did not show any specific trend in many groups.

In order to assess the impact of the policy and confirm the observations made, an ARIMAX model was utilized. This model considered the policy intervention as event inputs in the form of level-shift, ramp, and inverse trend to the previously fitted ARIMA models. Table 4 demonstrates that the policy intervention had a significant effect on reducing opioid consumption in specific groups: 03, 30, 11, 31, 13, and 23. However, there was no impact on other opioid classes or benzodiazepines. These results are noteworthy as the desired impact on classes 03 and 30, which represent 40% of the total transactions, may ultimately lead to the desired overall impact for opioid drugs. On the other hand, the highest volume prescription groups, namely classes 22 and 32, showed no desired policy effect.

Additionally, there was an unintended consequence of the policy on dispenser-isolated groups, specifically in classes 11 and 31. The unintended consequence is indicated by the positive coefficient of the exogenous policy covariate in these two classes, while in all other classes the coefficient is negative. This finding highlights the importance of carefully considering unintended consequences when implementing policies, especially in the healthcare industry.

Overall, the ARIMAX model provides a robust method for evaluating the impact of the policy intervention on prescription drug consumption. The results demonstrate the complexity of policy implementation and highlight the need for careful consideration of the potential consequences.

## 4. Discussion

The present study aimed to assess the impact of the opioid prescription limit legislation implemented in South Carolina. The results showed a significant reduction in overall opioid prescriptions, as indicated by the average daily MME decreasing from 53.68 (95% CI [53.33, 54.02]) to 51.09 (95% CI [50.74, 51.44]). These findings align with the intended purpose of opioid-limiting legislation, which is to reduce opioid exposure among patients and decrease the availability of opioids for misuse in the community [10,18]. However, the policy had minimal effect on benzodiazepine prescriptions, which were not the target of the policy. This underscores the need for policies that address the use of benzodiazepines, given the elevated risk of life-threatening overdose associated with their combination with opioids. Thus, the study emphasizes the importance of designing policies that account for the complexities of prescription drug use and misuse, as targeting only one drug may not suffice to address the larger issues at hand.

Moreover, recent policy revisions have recognized the significance of addressing benzodiazepines. For instance, starting January 2021, all controlled substances must be transmitted via electronic prescribing (S.C. Code Ann. § 44-53-360). Furthermore, starting April 2021, prescribers must offer naloxone prescriptions to patients if they concurrently prescribe an opioid and a benzodiazepine (S.C. Code Ann. § 44-53-361). However, there is still no direct policy to restrict the use of these drugs. These advancements are crucial and should be assessed using updated datasets to determine their efficacy in reducing the negative consequences associated with prescription drug use and abuse.

Economic research suggests that policies aimed at addressing the opioid crisis may have unintended consequences, such as an increase in harmful substance use from illicit markets [38]. Additionally, some state laws may inadvertently encourage opioid users to turn to the illicit market, leading to an increase in overdose rates and indicating that these types of legislation are not effective deterrents [14]. Our study also found mixed results for long-distance transactions after the policy was implemented in South Carolina, with patients receiving higher doses from prescribers located further away. This raises concerns about patients traveling longer distances to obtain prescriptions and incentivizing out-of-state procurement, which is consistent with our previous research that showed unintended shifts in the distribution of opioid drugs to neighboring states with more lenient regulations [23]. Collaborating among states to share information and track prescription data could help mitigate excessive opioid procurement, but policymakers should be aware of these unintended consequences.

Furthermore, our study indicates that different groups of patients classified based on the distance they travel to obtain their opioid prescriptions show distinct patterns of prescription levels. Particularly, it was found that patients who traveled distances exceeding 500 miles to obtain their drugs from distant pharmacies had a prescription volume higher than 90 MME per day, which set them apart from other groups. These findings align with earlier studies that suggest illegal activities such as doctor shopping are more prevalent across long distances and state borders [22,39]. In a future study, the underlying reasons for different prescription patterns based on patient characteristics such as age, gender, weight, pain level, prior opioid use, genetics, health status (including liver or kidney disease), and mental disorders like dual disorder status need to be investigated. The current data set lacks patient information, which makes it impossible to study these factors. It is important to consider these characteristics when prescribing opioids to ensure appropriate dosing and to minimize the risk of adverse effects [40]. In addition, it is important to study the impact of underlying disease conditions, such as a history of cancer, on opioid use in future research. Many cancer patients use opioids as part of palliative care to manage pain and improve their quality of life. However, prolonged opioid use can alter gene expression patterns, potentially leading to drug and therapy resistance [41].

Interestingly, our study also discovered that the current policy aimed at reducing opioid abuse and diversion is not effective in curbing this abnormal volume. We believe that an updated policy, one that puts more emphasis on regulating dispensers, is necessary to address this problem. This recommendation is reinforced by our observation that these groups have a higher proportion of retail and mail-order pharmacies compared to other groups. This is a significant finding since chain pharmacies are typically subject to more regulations while independent retail or mail-order pharmacies may have a greater likelihood of being linked to drug diversion due to their financial incentives and lack of information on patients’ prescription drug use history [42]. In light of these findings, policy makers should consider developing targeted interventions to address the specific issues associated with retail and mail-order pharmacies, such as improving monitoring and oversight mechanisms and increasing education and training for healthcare providers on the risks associated with these types of pharmacies. Nevertheless, it is crucial to note that further research is necessary to establish a causal relationship between the type of pharmacy and higher opioid consumption levels. Other potential causes for this phenomenon should also be explored.

The study had several limitations, including the lack of patient-level characteristics which hindered exploration of different prescription patterns among various groups. While the absence of impact on certain opioid classes does not imply policy failure, further research is necessary to understand the factors affecting the results, including patient and prescriber behavior. In particular, research is needed to investigate the impact of the policy on distant pharmacies and potentially illicit drugs. Future studies could also examine the impact of other policies and disruptions, such as COVID-19. To generalize the findings, exploring prescription data for other states is recommended. Additionally, studying the impact of such policies on opioid-related overdose deaths, while not the primary goal of the policy, would be valuable.

In conclusion, Our study demonstrates the usefulness of spatial classification of opioid prescriptions in understanding the variation in opioid prescription behavior and the impact of opioid prescription limit laws on different classes. Further research could investigate the reasons behind the lack of impact and explore ways to optimize the policy’s effectiveness for different opioid classes. By tailoring the policy to specific needs, policymakers can improve its impact and mitigate any unintended consequences. Overall, the authors recommend the following actions for policy makers:

1. To avoid potential unintended negative outcomes such as patients seeking opioids from distant pharmacies or turning to harmful illicit drugs, policymakers should carefully assess the impact of opioid-limiting legislation. This requires evaluating the effectiveness of policies in achieving their intended goals, while also considering their impact on different groups of patients, prescribers, and dispensers.
2. In particular, policymakers should examine the patterns of opioid consumption among different groups and explore the reasons for higher consumption levels among certain groups. Additionally, policymakers should take into account the characteristics of different types of pharmacies, such as independent retail or mail-order pharmacies, that may have a higher likelihood of being linked to drug diversion.
3. Furthermore, policymakers should consider the impact of prescription limit policies on other potentially harmful drugs, such as benzodiazepines, and evaluate the effectiveness of the policy in preventing overdoses. This evaluation should also take into account the impact of the policy on different types of transactions, particularly those that occur across state borders.

## 5. Conclusions

In conclusion, the study indicates that the prescription limitation law in South Carolina was effective in reducing overall opioid prescription volumes, but also revealed potential loopholes and unintended consequences of such policies. To address these issues, greater oversight of out-of-state doctors and regulations for mail-order and retail pharmacies may be necessary. Future research should examine the impact of prescription limitation laws on opioid-related harm and access to pain management for patients who require opioids. A multi-faceted approach is crucial to tackle the opioid crisis and promote safe and effective use of prescription opioids.

## Figures and Tables

**Table 1:** Classification system illustration.

Distance (Miles)	Disparity	Code	Distance (Miles)	Disparity	Code
π≤250	Patient isolated	**00**	500<π≤1000	Patient isolated	**20**
	Prescriber isolated	**01**		Prescriber isolated	**21**
	Dispenser isolated	**02**		Dispenser isolated	**22**
	Otherwise	**03**		Otherwise	**23**
250<π≤500	Patient isolated	**10**	π>1000	Patient isolated	**30**
	Prescriber isolated	**11**		Prescriber isolated	**31**
	Dispenser isolated	**12**		Dispenser isolated	**32**
	Otherwise	**13**		Otherwise	**33**

**Table 2:** Overview of key statistics in different coding system classes (opioid drugs only).

Group Code	Mean (SD) Days Supply	Mean (SD) MME	MME per Day (95% CI)	Percentage of MME
00	14.89 (4.87)	802.32 (310.46)	**48.88 (48.61, 49.15)**	12.05
01	15.05 (5.09)	771.95 (292.38)	**46.90 (46.70, 47.10)**	35.76
02	16.04 (4.91)	809.49 (304.97)	**48.66 (48.20, 49.12)**	3.87
03	15.4 (5.15)	800.95 (302.02)	**47.96 (47.75, 48.16)**	40.65
10	8.84 (3.74)	478.36 (362.17)	**44.00 (43.43, 44.57)**	0.19
11	14.89 (5.95)	815.39 (395.73)	**47.51 (47.15, 47.87)**	1.29
12	19.37 (7.62)	1064.53 (994.58)	**55.26 (53.66, 56.86)**	0.08
13	14.06 (5.20)	770.80 (357.95)	**49.08 (48.68, 49.47)**	1.66
20	7.56 (3.38)	309.30 (219.66)	**37.84 (37.44, 38.24)**	0.16
21	13.42 (6.25)	704.96 (411.89)	**46.40 (45.93, 46.93)**	0.80
22	18.93 (6.97)	1110.24 (746.99)	**91.71 (87.20, 96.22)**	0.07
23	12.69 (5.09)	721.36 (384.68)	**52.46 (51.91, 53.01)**	1.17
30	7.66 (3.15)	322.93 (230.59)	**37.78 (37.38, 38.18)**	0.19
31	12.69 (5.03)	723.15 (1336.44)	**47.50 (46.93, 48.07)**	1.22
32	20.06 (7.51)	1374.09 (1153.52)	**96.50 (93.43, 99.57)**	0.14
33	12.81 (4.67)	761.86 (375.38)	**56.00 (55.46, 56.54)**	1.87

**Table 3:** The MME per day in each group after and before policy implementation.

		Distance Level							
		Code 0X: π≤250		Code 1X: 250<π≤500		Code 2X: 500<π≤1000		Code 3X: π>1000	
		Mean		Mean		Mean		Mean	
		(95% CI)		(95% CI)		(95% CI)		(95% CI)	
		Pre-Policy	Post-Policy	Pre-Policy	Post-Policy	Pre-Policy	Post-Policy	Pre-Policy	Post-Policy
**Disparity Level**	**Code X0: Patient isolated**	50.57	46.84	47.38	39.93	40.41	34.74	40.01	35.10
		(50.25, 50.89)	(46.42, 47.26)	(46.58, 48.18)	(39.18, 40.68)	(39.95, 40.87)	(34.11, 35.37)	(39.45, 40.57)	(34.55, 35.65)
	**Code X1: Prescriber isolated**	49.18	44.15	48.78	45.98	48.56	43.81	46.69	48.49
		(48.91, 49.45)	(43.91, 44.39)	(48.38, 49.18)	(45.33, 46.63)	(48.03, 49.09)	(42.91, 44.71)	(46.35, 47.03)	(47.31, 49.67)
	**Code X2: Dispenser isolated**	49.14	48.08	58.43	51.92	85.07	99.76	97.10	95.78
		(48.69, 49.59)	(47.21, 48.95)	(55.87, 60.99)	(50.06, 53.78)	(80.84, 89.30)	(91.22, 108.30)	(93.36, 100.84)	(90.70, 100.86)
	**Code X3: Otherwise**	49.57	46.01	50.78	47.02	53.77	50.89	56.81	55.02
		(49.30, 49.84)	(45.73, 46.29)	(50.35, 51.21)	(46.34, 47.70)	(53.16, 54.38)	(49.93, 51.85)	(56.23, 57.39)	(54.05, 55.99)

**Table 4:** ARIMAX models for evaluating the policy impact on opioid drugs for classes with significant intervention coefficients.

*Class*	*Model*	*Coefficient*	*Estimate*	*Standard Error*	*p Value*	*Significance Level*
***Overall***	ARIMA (1,1,0)	*AR (12)*	0.45	0.10	p<0.001	(***)
		*Inverse Trend D(1)*	3241.52	1098.68	0.004	(**)
***11***	ARIMA (1,1,1)	*Constant*	−5.28	0.60	p<0.001	(***)
		*AR (12)*	−0.29	0.14	0.040	(*)
		*MA (1)*	1.00	0.03	p<0.001	(***)
		*Ramp*	−13.56	4.48	0.003	(**)
		*Level Shift*	11.97	2.29	p<0.001	(***)
***31***	ARIMA (1,2,2)	*Constant*	−5.38	2.66	0.046	(*)
		*MA (1)*	0.64	0.09	p<0.001	(***)
		*MA (12)*	0.85	0.11	p<0.001	(***)
		*Ramp*	−123.07	32.69	0.003	(***)
		*Level Shift*	21.57	6.51	0.002	(**)
***03***	ARIMA (1,2,2)	*MA (1)*	0.89	0.05	p<0.001	(***)
		*MA (12)*	−0.65	0.10	p<0.001	(***)
		*Level Shift*	−3.71	1.16	0.002	(**)
***30***	ARIMA (1,2,1)	*MA (12)*	0.69	0.09	p<0.001	(***)
		*Level Shift*	−63.04	6.54	p<0.001	(***)
***13***	ARIMA (1,1,1)	*MA (1)*	0.91	0.04	p<0.001	(***)
		*Level Shift*	−5.67	1.16	p<0.001	(***)
***23***	ARIMA (2,1,0)	*AR (1)*	0.46	0.10	p<0.001	(***)
		*AR (12)*	−0.64	0.11	p<0.001	(***)
		*Level Shift*	−41.73	19.19	0.032	(*)

Significance Codes: ***: 0.01, **: 0.05, *: 0.1.

## Data Availability

The data used in this study is based on the South Carolina Prescription Monitoring Program, which is provided by the South Carolina Department of Health and Environmental Control. This program is designed to monitor prescription drug use and provide healthcare providers with information to help prevent prescription drug abuse and diversion. However, the data is not publicly available.
